# Evaluation of acute oral toxicity of red alga (Gracilaria domingensis sonder ex kützing) in mice C57BL/6

**DOI:** 10.1186/1758-5996-7-S1-A252

**Published:** 2015-11-11

**Authors:** José Ytalo Gomes da Silva, Marcelo Oliveira Holanda, Carla Laine Silva Lima, Paula Alves Salmito Rodrigues, Erlândia Alves Magalhães Queiroz, Raquel Teixeira Terceiro Paim, Thais Vital de Freitas, Juliana Barbosa Dantas, Sandra Machado Lira, Natalia do Vale Canabrava, Mariana de Freitas Moreira, Bruno Bezerra Silva, Chayane Gomes Marques, Julianne do Nascimento Sales, Arnaldo Solheiro Bezerra, Emanuele Silva de Sousa, Rafaela Valesca Rocha Bezerra Sousa, Lia Magalhães de Almeida, Francisca Noélia Pereira Mendes, Icaro Gusmão Pinto Vieira, Maria Izabel Florindo Guedes

**Affiliations:** 1 Universidade Estadual do Ceará, Fortaleza, Brazil

## Background

Many species of plants have been used pharmacologically to treat the symptoms of diabetes mellitus. However, it is influenced by the toxicity of the plant extract used in the preparation, method of preparation and administration route, so it is important to identify potential risks regarding the toxicity of the product to be used. The red algae (Gracilaria domingensis) is a source of minerals, vitamins, fiber and low in lipids, and may contain anti-hyperglycemic activity, contributing thus in control of DM. Therefore, it becomes necessary to carry out acute toxicity tests to evaluate the red algae is safe for therapeutic use.

## Objective

To evaluate the acute toxicity of red algae in healthy mice.

## Materials and methods

A flour of algae was obtained by drying in an oven at 45 ° C with forced air circulation, followed by grinding. Twelve adult females C57BL/6 mice, weighing 22-25g, were used in this study. The experimental protocol of this study was submitted and accepted by the Ethics in Animal Research Committee (EARC) with number 90/10. The mice were divided in 2 groups (n=6) and fasted for 4 h. After this period, was administered by gavage, saline (1 mL/kg) to the control group, and the solution of algae at a dose of 2000 mg/kg to the test group, followed by behavioral observation of the animals at 30, 60, 90, 120, 150, 180, 210, 240, 270 and 300. After twelve days the animals were euthanized for removal and analysis of the relative weight vital organs to check for acute toxicity. The analysis of the significance of differences between the data was performed using nonparametric Mann Whitney, considering significant Results that had p <0.05.

## Results

**Figure 1:**
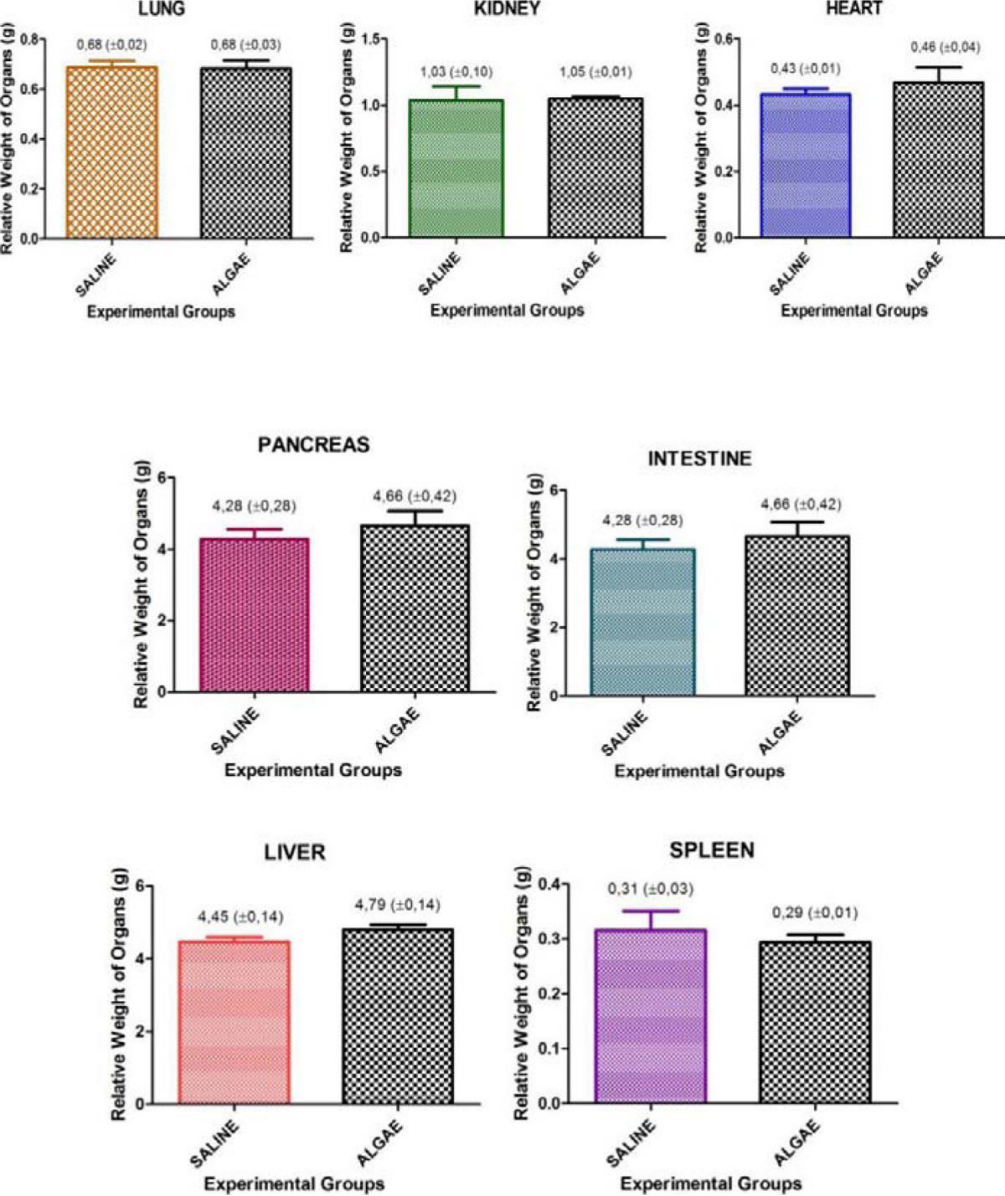
The relative weight of vital organs.

Deaths in the acute toxicity evaluation of red algae had not been registered in a dosage of 2000mg/kg in both groups, during the experiment. There were no significant changes in the Hippocratic screening, in macroscopic analysis of the organs, in physiological parameters, thus emphasizing the low toxicity of the algae solution. Also there was no statistically significant difference in analysis of the concerning weight vital organs, which confirms the low toxicity of the red alga (Figure 1).

## Conclusion

Given the above Results, it is concluded that the administered solution of the red algae does not have toxic effects and is safe for therapeutic use.

